# Automated pupillometry predicts neurological deterioration in the neurosurgical ICU: A retrospective observational study with secondary analysis in traumatic brain injury

**DOI:** 10.1097/MD.0000000000046809

**Published:** 2025-12-26

**Authors:** Zonghai Guo, Anyi Li, Yujing Liu, Pengfei Chang, Jie Cheng, Ran Zhou, Ying Yu, Ying Gao, Ran Zhao, Tengyu Che

**Affiliations:** a Department of Neurosurgery, The Affiliated Hospital of North China University of Science and Technology, Tangshan, Hebei, P.R. China.

**Keywords:** automated pupillometry, mediation analysis, neurosurgical intensive care, sedation status

## Abstract

The primary objective of this study was to test whether sedation depth mediates the association between pupillary reactivity, quantified by the Neurological Pupil index (NPi) and significant neurological deterioration in critically ill neurosurgical patients. A prespecified secondary objective was to explore whether this relationship differs between traumatic brain injury (TBI) and non-TBI diagnoses. We conducted a retrospective, single-center study of 360 adults admitted to a neurosurgical ICU (2019–2022) with daily automated pupillometry and detailed sedation records. “Significant deterioration” was defined as a ≥ 2-point decline in Glasgow Coma Scale, new focal deficits, or escalated neurosurgical intervention. The primary analysis used multivariable logistic regression and causal mediation (exposure: NPi; mediator: deep sedation, Richmond Agitation–Sedation Scale ≤ −3; outcome: deterioration; 5000 bootstrap samples), adjusting for diagnosis, admission GCS, hypertension, sedation agent and dose. A secondary stratified analysis compared TBI versus non-TBI. Of 360 patients, 80 (22.2%) deteriorated. Lower NPi and greater sedation intensity were independently associated with deterioration (per 1-point higher NPi: adjusted OR 0.73, 95% CI 0.59–0.91; deep sedation: adjusted OR 3.17, 95% CI 1.75–5.73). Higher admission GCS was protective (adjusted OR 0.88, 95% CI 0.81–0.95). Mediation analysis showed that sedation depth accounted for 19.6% of the total effect of lower NPi on deterioration (indirect effect β = 0.196; *P *< .01). In the secondary analysis, mediation was not significant for the TBI deterioration pathway (β = 0.08; *P *= .10), consistent with the dominant influence of structural injury severity in TBI. Lower pupillary reactivity is strongly associated with neurological deterioration, and this association is partly mediated by sedation depth. Clinically, trending NPi and individualizing sedation, especially when NPi is < 3.0 or declining may help mitigate secondary injury. In TBI, mediation by sedation depth was not evident, suggesting structural injury severity remains the principal determinant of decline.

## 1. Introduction

Pupillary reactivity has long been recognized as a key clinical indicator of neurological status in critically ill patients, reflecting both brainstem integrity and global cerebral function. Quantitative pupillometry, particularly the Neurological Pupil index (NPi), has emerged as a reliable, standardized approach to measuring pupillary reactivity in various acute brain injury settings.^[1–4]^ Lower NPi values have been associated with unfavorable neurological outcomes, including increased mortality, impaired functional recovery, and a higher propensity for long-term cognitive deficits.^[5,6]^ Despite its documented prognostic significance, the mechanisms by which pupillary reactivity correlates with clinical deterioration remain incompletely understood, particularly when confounded by critical care interventions such as sedation.^[7–9]^

Sedation is frequently employed in critically ill patients to facilitate mechanical ventilation, reduce agitation, and promote physiological stability.^[10–12]^ However, accumulating evidence suggests that sedation depth can independently influence neurological outcomes, especially when sedation is deep and prolonged. Excessive sedation has been correlated with delayed weaning from mechanical ventilation, prolonged ICU stay, and heightened risk of delirium.^[13–15]^ In neurocritical care specifically, sedation may obscure neurological examinations and potentially exacerbate secondary brain injury through alterations in cerebral metabolism and autoregulation.^[16–19]^ Given the pivotal role of pupillary reactivity in guiding clinical decisions, it is crucial to elucidate whether sedation depth serves merely as a confounder or functions as an actual mediator linking impaired pupillary reactivity to significant neurological deterioration.^[5,20]^

Traumatic brain injury (TBI) represents a distinct population where sedation is often necessary to manage intracranial pressure and prevent secondary insults.^[21]^ However, the severity of structural brain damage in TBI can overshadow the contribution of sedation depth, potentially reducing its role as a mediator in clinical outcomes.^[22,23]^ Limited research has directly examined how sedation interacts with pupillary reactivity to affect the risk of deterioration in a heterogeneous ICU population, including both TBI and non-TBI patients. Therefore, the primary objective of this study was to investigate whether sedation depth mediates the relationship between lower NPi and significant neurological deterioration, and whether such mediation differs between TBI and non-TBI cohorts. By clarifying these pathways, we aim to identify opportunities for more targeted and individualized sedation strategies to optimize neurological outcomes in critically ill patients.

## 2. Methodology

### 2.1. Study design and population

This was a retrospective, single-center study performed in the Neurosurgical Intensive Care Unit (NICU) of North China University of Science and Technology Affiliated Hospital from January 2019 to December 2022. We screened 420 adults (≥18 years) admitted with severe neurosurgical conditions – traumatic brain injury (TBI), subarachnoid hemorrhage (SAH), intracerebral hemorrhage (ICH), or brain tumor – requiring intensive monitoring, who had daily or more frequent automated pupillometry assessments.^[24–26]^ Patients lacking complete sedation or outcome data, discharged or deceased within 24 hours, or with ocular abnormalities preventing reliable pupillometry were excluded. A final sample of 360 patients was included.

### 2.2. Inclusion and exclusion criteria

Eligible patients were those aged ≥ 18 years, admitted primarily for an acute neurosurgical condition necessitating intensive care, receiving at least one pupillometry reading daily for ≥ 3 consecutive days, and having complete electronic medical records on sedation, pupillometry, and clinical outcomes. We excluded patients with any confounding ocular pathology, incomplete sedation records, or an ICU stay of < 24 hours.^[7,8,17,27]^ All prespecified inclusion and exclusion criteria were applied during data verification prior to any statistical analyses, yielding a primary analytic cohort of 360 patients.

### 2.3. Data collection

We extracted demographic data (age, sex), clinical variables (primary diagnosis, admission Glasgow Coma Scale [GCS], comorbidities), and sedation details (Richmond Agitation-Sedation Scale [RASS] category and midazolam or propofol with a calculated mean sedation dose in mg/kg/h).^[28]^ Automated pupillometry parameters (NPi, constriction velocity, minimum pupil diameter) were documented every 8 hours or upon clinical indication. For analysis, the mean or the most abnormal daily pupillometry reading was chosen to capture clinically relevant changes. All data were stored in a secured NICU registry and cross-referenced with electronic health records for accuracy. Sedation indications and targets varied by diagnosis. In TBI, deeper sedation could be deliberately titrated for intracranial pressure control at the attending physician’s discretion. In non-TBI conditions (SAH, ICH, brain tumors), sedation was typically used for ventilator synchrony, analgesia/anxiolysis, and to facilitate neurological examination; intracranial pressure-targeted deep sedation was applied only when clinically indicated. Protocols were not standardized at the study level; therefore, we captured sedation depth (RASS category), agent, and mean dose to reflect actual bedside practice.

### 2.4. Outcome definition

The primary outcome was “significant deterioration,” operationally defined as a drop of ≥ 2 points in GCS from baseline, development of new focal neurological deficits, or the need for an escalated neurosurgical intervention (decompressive craniectomy). All other patients were categorized as having “no significant deterioration.” Secondary outcomes included ICU length of stay, in-hospital mortality, the need for advanced airway or tracheostomy, and final discharge disposition.

### 2.5. Statistical analysis

All analyses were carried out using R (version 4.3.2). Continuous variables were described as mean ± standard deviation (or median with interquartile range when non-normal) and compared using *t* tests or Mann–Whitney *U* tests; categorical variables were presented as frequencies and compared by Chi-square or Fisher’s exact tests.^[29]^

To identify independent predictors of significant deterioration, we employed three nested multivariate logistic regression models: Model I: Adjusted for only basic demographic variables (age, gender); Model II: Further adjusted for primary neurosurgical concern, focusing on TBI versus other diagnoses due to its frequency and known impact; relevant comorbidity (hypertension) given its association with worse outcomes; clinical severity (admission GCS); sedation parameters (RASS-based category, sedation agent, mean sedation dose); and key pupillometry metric (mean NPi). These covariates were chosen based on clinical plausibility and prior literature; Model III: Retained only significant factors (*P *< .05) from Model II, giving a final parsimonious model.

For each covariate, we reported the adjusted odds ratio (OR) and 95% confidence interval (CI). The point estimates (Adjusted OR) lie near the geometric midpoint of the lower and upper CI on the log scale, confirming consistency in logistic modeling. Variables with negative coefficients (Estimate < 0) indicate a protective effect (they reduce the odds of deterioration).

Subsequently, we prespecified subgroup analyses stratified by primary diagnosis (TBI vs non-TBI), by sedation depth (RASS categories), and by the combined exposure of low pupillary reactivity (NPi < 3.0) with deep sedation. This TBI vs non-TBI dichotomization was chosen a priori because TBI constituted the largest and pathophysiologically distinct subgroup sedation in TBI is often titrated to manage intracranial pressure, whereas the remaining diagnoses (SAH, ICH, brain tumors) formed smaller, heterogeneous strata. Given 80 events in total, further splitting into multiple diagnosis-specific strata would have reduced events per variable and risked unstable or overfitted multivariable and mediation models. Accordingly, diagnosis-specific mediation models were not performed to preserve statistical stability and interpretability.

Finally, a mediation analysis was carried out using the “mediation” package in R to evaluate the potential mediating role of sedation depth (deep sedation vs others) on the relationship between NPi and deterioration risk, calculating total, direct, and indirect effects with 5000 bootstrapped iterations.^[30]^ Statistical significance was set at a two-tailed *P* value < .05.

### 2.6. Ethical considerations

All methods conformed to institutional ethics guidelines and the Declaration of Helsinki. This study was approved by the Institutional Review Board of the North China University of Science and Technology Affiliated Hospital (approval No. SQ20230718074). The Institutional Review Board deemed patient consent unnecessary due to the retrospective design. Patient confidentiality was preserved, and only deidentified data were used in the final analysis.

## 3. Results

A total of 360 patients were included in the final analysis, of whom 80 (22.2%) experienced significant deterioration. Table 1 presents the baseline characteristics of the study population, stratified by deterioration status. The mean age overall was 58.2 ± 15.7 years, and there was no statistically significant difference in age or sex distribution between those who deteriorated versus those who did not (*P* = .35 and *P* = .84, respectively). Patients in the deterioration group, however, more frequently presented with TBI (48.8% vs 32.5%, *P* = .009) and had a notably higher prevalence of hypertension (55.0% vs 37.1%, *P* = .004). Additionally, their admission Glasgow Coma Scale (GCS) score was significantly lower compared to the no-deterioration group (8.6 ± 3.2 vs 11.1 ± 2.9, *P* < .001). With respect to sedation parameters, patients in the deterioration group were more likely to be under deep sedation (75.0% vs 32.1%, *P* < .001), to have received midazolam as the sole sedative (37.5% vs 17.9%, *P *< .001), and to have a higher mean sedation dose (3.9 ± 1.5 vs 2.8 ± 1.2 mg/kg/h, *P *< .001). Pupillometry findings also differed: those with significant deterioration had a lower mean NPi (2.8 ± 1.2 vs 3.6 ± 1.0, *P *< .001) and reduced maximum constriction velocity. Furthermore, the deterioration group had an extended NICU stay (12.6 ± 5.1 vs 8.6 ± 3.9 days, *P *< .001).

**Table 1:** Baseline characteristics of neurosurgical ICU patients with and without significant neurological deterioration.

Variables	Total (n = 360)	No significant deterioration (n = 280)	Significant deterioration (n = 80)	*P* value
Age (mean (SD), years)	58.2 (15.7)	57.8 (16.3)	59.4 (14.2)	.35
Gender (n, %)				
Male	215 (59.7)	168 (60.0)	47 (58.8)	.84
Female	145 (40.3)	112 (40.0)	33 (41.2)	.84
Primary diagnosis (n, %)				
Traumatic brain injury (TBI)	130 (36.1)	91 (32.5)	39 (48.8)	.009
Subarachnoid hemorrhage (SAH)	90 (25.0)	72 (25.7)	18 (22.5)	.54
Intracerebral hemorrhage (ICH)	80 (22.2)	72 (25.7)	8 (10.0)	.002
Brain tumor	60 (16.7)	45 (16.1)	15 (18.7)	.52
Comorbidities (n, %)				
Hypertension	148 (41.1)	104 (37.1)	44 (55.0)	.004
Diabetes mellitus	70 (19.4)	49 (17.5)	21 (26.3)	.07
Admission GCS (mean (SD))	10.5 (3.1)	11.1 (2.9)	8.6 (3.2)	<.001
Sedation (RASS-based) (n, %)				
Deep sedation (RASS ≤ −3)	150 (41.7)	90 (32.1)	60 (75.0)	<.001
Light/moderate sedation (−2 to 0)	110 (30.6)	100 (35.7)	10 (12.5)	<.001
Minimal/no sedation (RASS ≥ +1)	100 (27.8)	90 (32.1)	10 (12.5)	<.001
Sedation agent (n, %)				
Propofol only	120 (33.3)	95 (33.9)	25 (31.3)	.67
Midazolam only	80 (22.2)	50 (17.9)	30 (37.5)	<.001
Combination/ other	160 (44.4)	135 (48.2)	25 (31.3)	.008
Mean sedation dose* (mean (SD), mg/kg/h)	3.1 (1.4)	2.8 (1.2)	3.9 (1.5)	<.001
Pupillometry				
Mean NPi (mean (SD))	3.4 (1.1)	3.6 (1.0)	2.8 (1.2)	<.001
Max constriction velocity (mean (SD), mm/s)	2.2 (0.8)	2.4 (0.7)	1.7 (0.6)	<.001
Min pupil diameter (mean (SD), mm)	2.1 (0.6)	2.2 (0.6)	1.7 (0.4)	<.001
Length of NICU stay (days, mean (SD))	9.5 (4.2)	8.6 (3.9)	12.6 (5.1)	<.001

GCS = Glasgow Coma Scale, NICU = Neurosurgical Intensive Care Unit, NPi = Neurological Pupil index, RASS = Richmond Agitation-Sedation Scale.

* Mean Sedation Dose refers to averaged total sedative infusion rate (e.g., propofol or midazolam) recorded daily during NICU stay.

**Figure 1.**
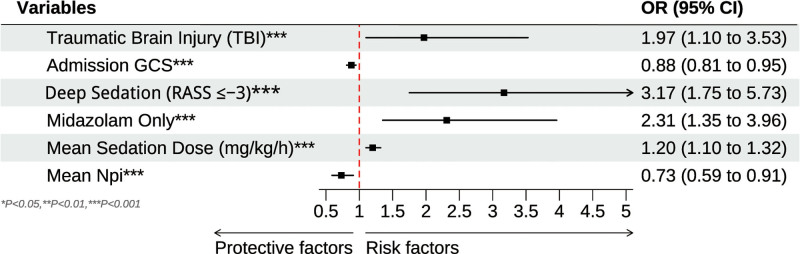
The forest plot of significant influencing factors for significant deterioration in neurosurgical ICU patients after adjusting for all confounders.

Table 2 summarizes the results of the multivariate logistic regression analyses. Model I, adjusting for age and gender only, did not identify any significant predictors of deterioration. In Model II, which incorporated TBI, hypertension, admission GCS, sedation depth and agent, mean sedation dose, and mean NPi, several factors emerged as significant. TBI (Adjusted OR = 2.11, 95% CI: 1.17–3.82, *P *= .013), deep sedation (Adjusted OR = 3.32, 95% CI: 1.88–5.86, *P* < .001), midazolam use (Adjusted OR = 2.51, 95% CI: 1.44–4.36, *P *= .001), higher mean sedation dose (Adjusted OR = 1.22 per mg/kg/h, 95% CI: 1.11–1.34, *P *< .001), and lower mean NPi (Adjusted OR = 0.68, 95% CI: 0.55–0.86, *P *= .001) were each independently linked to a greater likelihood of significant deterioration. Model III retained the predictors that remained statistically significant (*P* < .05) from Model II. Traumatic brain injury (Adjusted OR = 1.97, 95% CI: 1.10–3.53, *P* = .022), deep sedation (Adjusted OR = 3.17, 95% CI: 1.75–5.73, *P* < .001), midazolam alone (Adjusted OR = 2.31, 95% CI: 1.35–3.96, *P* = .002), higher mean sedation dose (Adjusted OR = 1.20, 95% CI: 1.10–1.32, *P *< .001), and lower mean NPi (Adjusted OR = 0.73, 95% CI: 0.59–0.91, *P *= .005) all remained significant, underscoring their critical role in predicting deterioration. Admission GCS (Adjusted OR = 0.88, 95% CI: 0.81–0.95, *P* = .002) also showed a protective effect, indicating that patients with higher initial GCS scores were less likely to deteriorate. Figure 1 shows the forest plot of significant influencing factors for significant deterioration in neurosurgical ICU patients based on Model III.

**Table 2:** Multivariable logistic regression of factors associated with significant neurological deterioration in neurosurgical ICU patients.

Variables	Estimate	Standard error	Adjusted OR*	Lower CI*	Upper CI*	*P* value
Model I						
Age (years)	0.02	0.01	1.02	1.00	1.04	.09
Male	0.07	0.14	1.07	0.81	1.41	.62
Model II						
Age (years)	0.01	0.01	1.01	0.99	1.03	.29
Male	0.05	0.13	1.05	0.80	1.38	.74
Traumatic brain injury (TBI)	0.74	0.30	2.11	1.17	3.82	.013
Hypertension	0.48	0.25	1.62	1.00	2.60	.050
Admission GCS	−0.15	0.04	0.86	0.79	0.94	.001
Deep sedation (RASS ≤ −3)	1.20	0.30	3.32	1.88	5.86	<.001
Midazolam only	0.92	0.28	2.51	1.44	4.36	.001
Mean sedation dose (mg/kg/h)	0.20	0.05	1.22	1.11	1.34	<.001
Mean NPi	−0.38	0.12	0.68	0.55	0.86	.001
Model III						
Traumatic brain injury (TBI)	0.68	0.29	1.97	1.10	3.53	.022
Admission GCS	−0.13	0.04	0.88	0.81	0.95	.002
Deep sedation (RASS ≤ −3)	1.15	0.31	3.17	1.75	5.73	<.001
Midazolam only	0.84	0.27	2.31	1.35	3.96	.002
Mean sedation dose (mg/kg/h)	0.18	0.05	1.20	1.10	1.32	<.001
Mean NPi	−0.32	0.11	0.73	0.59	0.91	.005

Negative coefficients (estimate < 0) represent a reduction in the probability of deterioration for each unit increase in the respective variable.

• Model I: Adjusted for age, gender.

• Model II: Further adjusted for traumatic brain injury (TBI), hypertension, admission GCS, sedation category, sedation agent, mean sedation dose, and mean NPi.

• Model III: Includes statistically significant factors retained from Model II.

Adjusted OR = adjusted odds ratio, 95% CI = 95% confidence interval, GCS = Glasgow Coma Scale, NPi = Neurological Pupil index, RASS = Richmond Agitation-Sedation Scale.

* Adjusted OR and confidence interval derived from the multivariable logistic regression model.

Prespecified subgroup results are presented in Table 3. Compared with minimal/no sedation, deep sedation was associated with a higher prevalence of significant deterioration (54.3% vs 15.5%, *P *< .001). When stratified by diagnosis, deep sedation conferred greater odds of deterioration in TBI (OR 3.96, 95% CI 2.05–7.65; *P *< .001) and remained significant in non-TBI (OR 2.41, 95% CI 1.24–4.68; *P* = .009). Among patients with NPi < 3.0 who were deeply sedated, the odds were highest (OR 4.88, 95% CI 2.28–10.44; *P *< .001). We did not present additional diagnosis-specific mediation models (SAH, ICH, brain tumors) because subgroup event counts were insufficient for reliable multivariable estimation within this cohort.

**Table 3:** Prespecified subgroup analyses of significant neurological deterioration.

Subgroup/comparison	Definition of exposure (reference)	OR (95% CI)	*P* value
Diagnosis-stratified effect of deep sedation			
TBI only	Deep sedation (RASS ≤ −3) vs minimal/no sedation (RASS ≥ +1)	3.96 (2.05–7.65)	<.001
Non-TBI only	Deep sedation (RASS ≤ −3) vs minimal/no sedation (RASS ≥ +1)	2.41 (1.24–4.68)	.009
Low NPi combined with deep sedation	NPi < 3.0 and deep sedation vs NPi ≥ 3.0 and not deep sedation	4.88 (2.28–10.44)	<.001
Overall event rates by sedation depth	Deep sedation (RASS ≤ −3) vs minimal/no sedation (RASS ≥ +1)	-	<.001

Odds ratios estimated within the indicated subgroups using logistic regression consistent with the main analysis definitions.

CI = confidence interval, NPi = Neurological Pupil index, OR = odds ratio, RASS = Richmond Agitation–Sedation Scale, TBI = traumatic brain injury.

**Figure 2.**
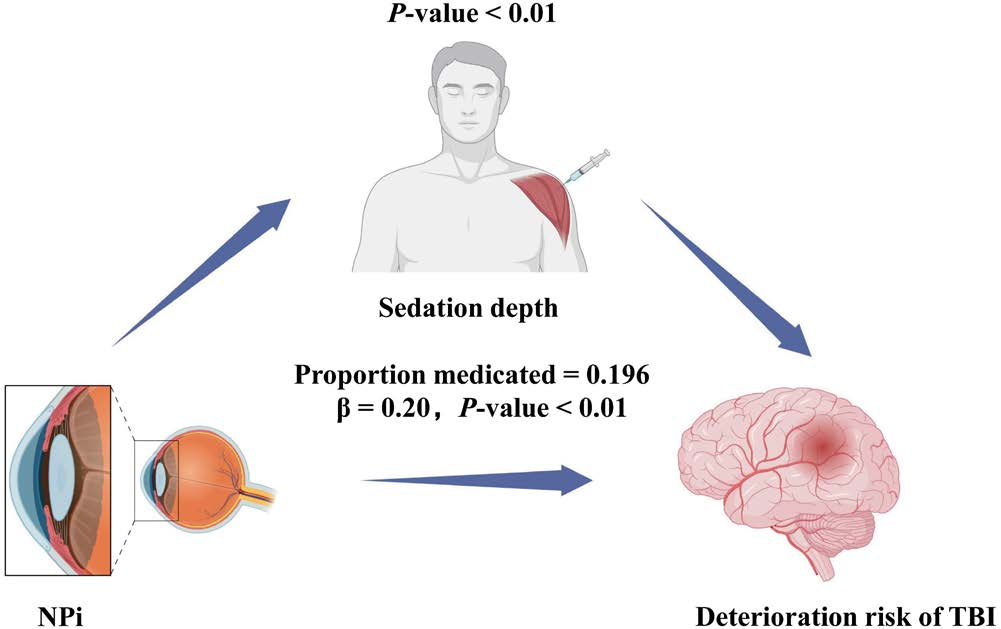
Path diagram of mediation analysis of relationship between NPi, sedation depth and deterioration risk of TBI. NPi = neurological pupil index, TBI = traumatic brain injury.

A mediation analysis was performed to explore whether sedation depth (deep sedation vs lighter/none) mediated the relationship between pupillary reactivity and significant deterioration. In particular, mean NPi was tested as the predictor, sedation depth as the mediator, and significant deterioration as the outcome. Figure 2 illustrates the path diagram of this model. Sedation depth accounted for 19.6% of the total effect of lower NPi on significant deterioration (indirect effect: β = 0.196, *P* < .01), indicating that reductions in pupillary reactivity partially increased deterioration risk through its association with deeper sedation requirements. However, sedation depth was not a significant mediator in the relationship between TBI and deterioration (indirect effect: β = 0.08, *P* = .10). Thus, sedation depth significantly mediated the association between lower NPi and deterioration at the cohort level (and within non-TBI), but it did not mediate the pathway from TBI status to deterioration.

To assess robustness, we repeated the primary logistic regression models on reduced samples. First, we re-ran the models after additionally excluding 30 patients with incomplete pupillometry series or sedation-protocol changes within the first 24 hours (n = 330). Second, in a more conservative specification, we also excluded 15 patients with retrospectively documented major ocular injuries or preexisting pupillary abnormalities (n = 315). These exclusions were applied only for sensitivity analyses and did not affect the primary cohort (n = 360). Across both specifications, the direction and magnitude of the key associations (including mean NPi, sedation depth and TBI) were unchanged, with <10% variation in adjusted odds ratios.

## 4. Discussion

Our study aimed to investigate whether sedation depth mediates the relationship between pupillary reactivity and significant neurological deterioration in critically ill patients. Our findings indicate that lower NPi is associated with an increased risk of significant deterioration. Mediation analysis demonstrated that deeper sedation partially accounted for this association, explaining 19.6% of the total effect. This suggests that reduced pupillary reactivity not only serves as an indicator of impaired neurological function but may also indirectly contribute to worse outcomes by necessitating more profound sedation. However, in patients with TBI, sedation depth did not significantly mediate the relationship between injury and deterioration, indicating that the primary driver of neurological decline in this population is the severity of the structural brain damage itself.

Our findings align with previous studies demonstrating the prognostic value of pupillary reactivity in critically ill patients.^[7,23,31–33]^ The NPi has been widely recognized as a reliable neurological assessment tool, with previous studies indicating that lower NPi values are predictive of poor neurological outcomes, including increased mortality and unfavorable functional recovery.^[34,35]^ Similarly, the relationship between deeper sedation and adverse neurological outcomes has been reported in studies on critically ill patients undergoing mechanical ventilation, where excessive sedation has been linked to delayed recovery and increased mortality.^[36,37]^ However, our study provides a novel perspective by quantitatively assessing the mediating role of sedation depth in the pathway from reduced pupillary reactivity to neurological deterioration, highlighting a potentially modifiable factor in patient management.

Mediation analysis demonstrated that sedation depth explained a significant proportion of the association between lower NPi and deterioration, supporting the hypothesis that patients with reduced pupillary reactivity may require deeper sedation, which in turn contributes to worse outcomes. This is in line with prior work,^[38–40]^ which found that sedation protocols significantly impact neurological recovery, especially in patients with acute brain injuries. However, in the case of TBI, our study did not identify sedation depth as a significant mediator. This could be due to the fact that in TBI patients, the severity of structural brain injury, rather than sedation per se, primarily dictates neurological outcomes. Similar findings were reported by another study,^[14,18,41]^ which emphasized that TBI management should focus more on intracranial pressure control and neuroprotection rather than sedation modulation. In TBI, sedation depth may reflect a proactive, protocol-driven strategy for intracranial pressure control rather than worsening neurological function per se. By contrast, in non-TBI conditions (SAH, ICH, brain tumors), sedation is typically titrated for ventilator synchrony, analgesia/anxiolysis, and to facilitate examination, so greater sedation depth may more directly track neurological vulnerability. Within this context, NPi retains prognostic value across diagnoses, with both absolute thresholds (NPi < 3.0) and downward trends signaling elevated risk, especially when co-occurring with deep sedation.^[11,12,40]^

We contrasted TBI with other neurosurgical conditions a priori because TBI is pathophysiologically distinct and was the largest stratum in our dataset, while further dividing the non-TBI group (SAH, ICH, brain tumors) would have underpowered multivariable and mediation analyses and inflated multiplicity. Within this framework, sedation depth significantly mediated the pathway from lower NPi to deterioration in the overall/non-TBI cohorts but not along the TBI–deterioration pathway, an observation that is biologically plausible given that structural injury severity typically dominates prognosis in TBI. We acknowledge potential heterogeneity within the non-TBI group and note that larger, disease-specific cohorts are needed to test whether mediation magnitudes differ across SAH, ICH and brain tumors.

Subgroup analysis showed that the impact of sedation depth was more pronounced in the non-TBI cohort, whereas in TBI patients the direct effect of injury on deterioration remained predominant. These results suggest that in non-TBI populations, optimizing sedation strategies may offer an avenue for improving neurological outcomes. Previous studies have similarly shown that in medical ICU patients without focal brain injury, excessive sedation can exacerbate delirium and cognitive dysfunction, further increasing the risk of adverse outcomes.^[42]^ One possible explanation is that sedation in non-TBI patients may directly influence neuronal network activity and cerebral autoregulation, thereby modulating the trajectory of neurological recovery. In contrast, in TBI patients, the predominant drivers of deterioration are mechanical and vascular injury-related processes, which are less affected by sedation depth.^[4,43]^

We do not imply that subarachnoid hemorrhage, intracerebral hemorrhage and brain tumors share identical mechanisms or treatments; they were grouped as “non-TBI” to preserve statistical stability given event counts. Sedation indications and targets differ across these diagnoses, which may partly explain variation in the observed mediation effect. The absence of a unit-level standardized sedation protocol and the heterogeneity within the non-TBI group may introduce residual confounding; future studies with larger, disease-specific cohorts should test whether mediation magnitudes differ across SAH, ICH and brain tumors.

From a pathophysiological perspective, deep sedation may exacerbate neurological deterioration through multiple mechanisms. Prolonged sedation has been associated with impaired cerebral autoregulation, decreased synaptic plasticity, and neuronal apoptosis, all of which contribute to worse neurological recovery.^[2,44]^ Furthermore, anesthetic agents used for sedation, such as propofol and benzodiazepines, have been shown to alter mitochondrial function and neurotransmitter homeostasis, potentially worsening neuronal injury in already vulnerable patients.^[45]^ The finding that sedation depth mediates the association between NPi and deterioration suggests that deep sedation may contribute to secondary neurological injury, possibly through hypoperfusion-induced metabolic dysregulation and delayed neuronal repair mechanisms. This highlights the need for more individualized sedation strategies, particularly in patients with altered pupillary reactivity.

Our study has several strengths. We employed a robust mediation analysis framework to dissect the complex interplay between pupillary reactivity, sedation depth, and neurological deterioration. Additionally, our study contributes to a growing body of literature highlighting the impact of sedation strategies on neurological outcomes in critically ill patients. However, some limitations must be acknowledged. First, this was an observational study, limiting our ability to infer causality. Second, sedation protocols were not standardized across all patients, potentially introducing variability in sedation depth assessment. Third, while NPi is an objective measure of pupillary reactivity, other neurological assessment tools such as electroencephalography or brainstem reflexes were not included, which might have provided a more comprehensive picture of neurological function. Future studies should explore interventional approaches to optimize sedation depth based on real-time neurological monitoring to improve patient outcomes.

## 5. Conclusion

Our study highlights the complex interplay between pupillary reactivity, sedation depth, and neurological deterioration in critically ill patients. Our findings demonstrate that lower Neurological Pupil index is associated with an increased risk of deterioration, with sedation depth partially mediating this relationship. However, in traumatic brain injury patients, sedation depth did not significantly mediate deterioration, suggesting that structural brain injury severity remains the primary determinant of outcomes in this population. These results underscore the need for careful sedation management, particularly in patients with impaired pupillary reactivity, to minimize the potential for secondary neurological injury. Future research should explore personalized sedation strategies integrating real-time neurological monitoring to optimize patient outcomes.

## Acknowledgments

The views expressed in this publication are those of the authors.

## Author contributions

**Conceptualization:** Zonghai Guo.

**Data curation:** Anyi Li.

**Formal analysis:** Yujing Liu.

**Funding acquisition:** Zonghai Guo.

**Investigation:** Pengfei Chang.

**Methodology:** Jie Cheng.

**Project administration:** Ran Zhou.

**Resources:** Ying Yu.

**Software:** Ying Gao.

**Supervision:** Ran Zhao.

**Writing – original draft:** Zonghai Guo, Tengyu Che.

**Writing – review & editing:** Zonghai Guo, Tengyu Che.
